# Calmodulin kinase II initiates arrhythmogenicity during metabolic acidification in murine hearts

**DOI:** 10.1111/j.1748-1716.2009.01991.x

**Published:** 2009-09

**Authors:** T H Pedersen, I S Gurung, A Grace, C L-H Huang

**Affiliations:** 1 Physiological Laboratory, University of CambridgeCambridge, UK; 2 Department of Biochemistry, University of CambridgeCambridge, UK; 3 Department of Physiology and Biophysics, Aarhus UniversityAarhus, Denmark

**Keywords:** action potentials, arrhythmia, CaMKII, electrophysiology, metabolic acidosis

## Abstract

**Aim:**

The multifunctional signal molecule calmodulin kinase II (CaMKII) has been associated with cardiac arrhythmogenesis under conditions where its activity is chronically elevated. Recent studies report that its activity is also acutely elevated during acidosis. We test a hypothesis implicating CaMKII in the arrhythmogenesis accompanying metabolic acidification.

**Methods:**

We obtained monophasic action potential recordings from Langendorff-perfused whole heart preparations and single cell action potentials (AP) using whole-cell patch-clamped ventricular myocytes. Spontaneous sarcoplasmic reticular (SR) Ca^2+^release events during metabolic acidification were investigated using confocal microscope imaging of Fluo-4-loaded ventricular myocytes.

**Results:**

In Langendorff-perfused murine hearts, introduction of lactic acid into the Krebs-Henseleit perfusate resulted in abnormal electrical activity and ventricular tachycardia. The CaMKII inhibitor, KN-93 (2 μm), reversibly suppressed this spontaneous arrhythmogenesis during intrinsic rhythm and regular 8 Hz pacing. However, it failed to suppress arrhythmia evoked by programmed electrical stimulation. These findings paralleled a CaMKII-independent reduction in the transmural repolarization gradients during acidosis, which previously has been associated with the re-entrant substrate under other conditions. Similar acidification produced spontaneous AP firing and membrane potential oscillations in patch-clamped isolated ventricular myocytes when pipette solutions permitted cytosolic Ca^2+^ to increase following acidification. However, these were abolished by both KN-93 and use of pipette solutions that held cytosolic Ca^2+^ constant during acidosis. Acidosis also induced spontaneous Ca^2+^ waves in isolated intact Fluo-4-loaded myocytes studied using confocal microscopy that were abolished by KN-93.

**Conclusion:**

These findings together implicate CaMKII-dependent SR Ca^2+^ waves in spontaneous arrhythmic events during metabolic acidification.

Previous experiments in isolated whole hearts (Eloff *et al.* 2003), strips of cardiac tissue (Coetzee & Opie 1987, Orchard *et al.* 1987) and single cardiomyocytes (Kurachi 1982, Coetzee & Opie 1987) have consistently demonstrated marked arrhythmogenic effects during cell acidification (Orchard & Cingolani 1994). Acidification is an early and important biochemical change associated with acute myocardial ischaemia (Elliott *et al.* 1992, Vandenberg *et al.* 1993) and may contribute importantly to the enhanced risks of potentially life-threatening arrhythmias in such situations (Senges *et al.* 1979, Luqman *et al.* 2007). The mechanisms underlying arrhythmogenicity in metabolic acidification have therefore attracted extensive study (Orchard & Cingolani 1994).

More recently, acidification has also been associated with elevated activity in the multifunctional, Ca^2+^-dependent signal molecule calmodulin kinase II (CaMKII) (Hulme *et al.* 1997, Komukai *et al.* 2001, DeSantiago *et al.* 2004). CaMKII has been previously shown to exert generally pro-arrhythmic effects (Wu *et al.* 2002, Anderson 2007) under conditions of its *chronic* elevation. For example, its overexpression increases arrhythmogenic tendency by causing cardiac hypertrophy in murine hearts (Wu *et al.* 2002, Zhang *et al.* 2003, Kohlhaas *et al.* 2006). It has also been implicated in arrhythmogenesis in atrial natriuretic peptide-deficient mice (Kirchhof *et al.* 2004). However, CaMKII may also be involved in *acute* regulation of cardiac function (Komukai *et al.* 2001, Kohlhaas *et al.* 2006). Thus, inotropic effects leading to recovery of contractile function in ventricular myocytes during sustained acidification are blocked by the widely used and specific CaMKII inhibitor, KN-93 (Sumi *et al.* 1991, DeSantiago *et al.* 2004). This inotropic effect was attributed to increased SR Ca^2+^ reuptake and SR Ca^2+^ content due to a CaMKII-specific phosphorylation of phospholamban and consequent restoration of action potential (AP)-evoked Ca^2+^ transients (Komukai *et al.* 2001, DeSantiago *et al.* 2004). Thus, acidosis significantly increased the amplitude of the caffeine-induced Ca^2+^ transient used to assess SR Ca^2+^ content, but this increase with acidosis did not take place in the presence of KN-93 (Komukai *et al.* 2001). However, the accompanying elevation of SR Ca^2+^ content with such acidosis could potentially also cause SR Ca^2+^ overload leading to spontaneous SR Ca^2+^ release, spontaneous APs and triggered arrhythmias (Dibb *et al.* 2007), all features also associated with acidification (Orchard *et al.* 1987, Orchard & Cingolani 1994). Taken together these studies implicate CaMKII in the arrhythmia associated with acidosis. This notion is further strengthened by a recent study reporting that after a period of respiratory acidosis a CaMKII-dependent elevation in the SR Ca^2+^ content/leak induced enhanced triggering of spontaneous APs (Said *et al.* 2008). The role of CaMKII for arrhythmia arising *during* acidosis and particularly during metabolic acidosis is, however, unknown. Yet the above findings raise the hitherto untested possibility that CaMKII is also involved in the enhanced arrhythmogenic properties during metabolic acidosis. The present study accordingly investigated for possible acute roles of CaMKII in the arrhythmogenicity during metabolic acidification in murine hearts for the first time. Using Langendorff-perfused hearts we first associated metabolic acidification with enhanced arrhythmic substrate and triggered arrhythmic events. We then demonstrated that CaMKII inhibition by KN-93 reduced the incidence of spontaneous arrhythmias during metabolic acidification in Langendorff-perfused hearts. Finally, in single ventricular myocytes we show that two independent approaches for CaMKII inhibition markedly suppressed electrical abnormalities and Ca^2+^ waves during metabolic acidification.

## Materials and methods

### Animal handling and heart isolation

All animal handling followed UK animal welfare regulations. Mice were fed *ad libitum* and kept under a constant temperature environment with 12 : 12 h light : dark cycles. Wild-type male or female mice (age 3–7 months) mice were killed by cervical dislocation (Schedule 1, UK Animals (Scientific Procedures) Act 1986). Hearts were rapidly excised, submerged under ice-cold Krebs-Henseleit solution and the aorta cannulated and fixed to a size 21-gauge cannula using a micro-aneurysm clip (Harvard Apparatus, Edenbridge, UK).

### MAP recordings from Langendorff-perfused hearts

Cannulated hearts were retrogradely perfused in a Langendorff perfusion set-up with a flow rate of 2–3 mL min^−1^. The flow rate was kept constant using a peristaltic pump (101U/R; Watson Marlow, Falmouth, UK). The heated perfusate (37 °C) was passed through a 2 μm filtering membrane (Millipore, Watford, UK). The control Krebs-Henseleit solution contained (in mm): 119 NaCl, 25 NaHCO_3_, 4.0 KCl, 1.2 KH_2_PO_4_, 1.0 MgCl_2_, 1.8 CaCl_2_, 10 glucose, and 2.0 sodium pyruvate. In the solution designed to cause metabolic acidification, 20 mm NaHCO_3_ was exchanged with 20 mm sodium l-lactate. During all experiments, solutions were continuously bubbled using 95%/5% O_2_/CO_2_ resulting in pH ∼7.4 for control solutions and ∼6.7 for solutions causing metabolic acidification.

Cardiac electrical activity was recorded from the epicardial or endocardial surface of the left ventricle using unipolar monophasic action potential (MAP) electrodes (Linton Instruments, Harvard Apparatus, Edenbridge, UK). Recordings were bandpass filtered (0.005–1 kHz) and sampled at 5 kHz using a Neurolog amplifier (NL-900D; Digitimer, Welwyn Garden City, UK), a 1401 *plus* interface and spike software (Cambridge Electronic Design, Cambridge, UK). Electrical activity was recorded from hearts during three types of stimulation: (1) intrinsic rhythm, (2) regular 8 Hz pacing and (3) programmed electrical stimulation (PES) (Killeen *et al.* 2007, Thomas *et al.* 2008). The latter two types of stimulation excited the right ventricle using constant voltage pulses (1–4 V, 2 ms duration; Grass S48 stimulator, Grass Technologies, West Warwick, RI, USA). PES stimulation mimics the appearance of triggered activity and therefore permits assessment for the presence or otherwise of arrhythmic substrate.

### Isolation of ventricular myocytes

Cannulated hearts were retrogradely perfused at 2–3 mL min^−1^ (37 °C) in a Langendorff set-up similar to that used for MAP recordings. All solutions used in the isolation procedure and for storing isolated myocytes during experiments were made from the same basic solution containing (in mm): 120 NaCl, 5.4 KCl, 5 MgSO_4_, 10 glucose, 5.4 sodium pyruvate, 20 taurine and 10 HEPES. During the first 2 min of the isolation procedure, hearts were perfused with a nominally Ca^2+^-free solution (pH 6.95), made from the basic solution but also containing 5 mm NTA (*N*,*N*-bis[carboxymethyl]glycine disodium salt, Ca^2+^ chelator) intended to sequester extracellular Ca^2+^. Subsequently, hearts were perfused for 10 min with a digestion solution modified from the basic solution by additionally containing 250 μm CaCl_2_, collagenase type II (13 000 units in 50 mL) and hyaluronidase (22 000 units in 50 mL), pH 7.4. The ventricles were then isolated and placed in a modified basic solution that additionally contained 250 μm CaCl_2_, 2 mg mL^−1^ BSA and pH 7.4 to stop enzymatic digestion. Ventricles were then gently cut and triturated using disposable Pasteur pipettes to separate the myocytes. After 15–20 min rest the myocytes were transferred to the storing solution. This was essentially the basic solution but additionally contained 1 mg mL^−1^ BSA and 1 mm CaCl_2_.

### Patch clamping experiments

Single cell APs were recorded using whole-cell patch-clamping in the current clamp mode (Axoclamp 700B, Molecular Devices, Sunnyvale, CA, USA). Pipettes (2–4 MΩ) were pulled from filamented borosilicate glass (GC150F-10; Harvard Apparatus) using a vertical pipette puller (PC-10; Narishige, Tokyo, Japan). Myocytes were placed in a perfusion chamber (PDMI-2, Harvard Apparatus) that allowed a complete solution change within ∼2 min (flow rate ∼2 mL min^−1^, gravity driven) and visualization of myocytes was achieved using an inverted microscope (Nikon Eclipse, TE2000-U; Neurolog, Welwyn Garden City, UK). All recordings were performed at 37 °C. All recordings were performed without applying holding current and APs were triggered by applying small constant current pulses through the recording electrode. Recordings were low-pass filtered at 10 kHz and sampled at 20 kHz. The external control solution contained (in mm): 145 NaCl, 5 KCl, 10 HEPES, 10 glucose, 1 MgCl_2_, 1 CaCl_2_; pH was adjusted to 7.4 with NaOH. In solutions designed to produce metabolic acidification, 20 mm NaCl was replaced by 20 mm sodium l-lactate, and pH was adjusted to 6.7 with NaOH.

In the patch-clamp experiments two pipette solutions were used. In one solution a rise in cytosolic free Ca^2+^ during acidification was prevented by using 5 mm BAPTA as the Ca^2+^ chelating agent (BAPTA solution) (Tsien 1980). In contrast to BAPTA, the Ca^2+^ chelating property of EGTA is highly pH sensitive in the physiological pH range (Harrison & Bers 1989). Accordingly, in the second solution both BAPTA and EGTA were included in such proportions (EGTA-BAPTA solution, 5 mm total) that during acidosis the change in free Ca^2+^ could replicate that occurring in acidified, intact myocytes. In both solutions the free Ca^2+^ was 50 nm at pH 7.2. When acidified, the BAPTA solution could be assumed to maintain the free Ca^2+^ constant (Tsien 1980) while in the EGTA-BAPTA solution the free Ca^2+^ would increase from ∼50 to ∼300 nm with a drop of pH from 7.2 to 6.7. The proportions of EGTA and BAPTA in the EGTA-BAPTA solution were estimated as follows. The total Ca^2+^ in a solution containing both EGTA and BAPTA at pH 7.2 and 6.7, respectively, is given by: 
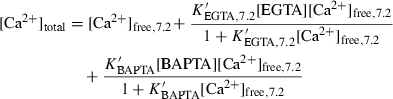

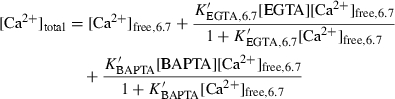


Assuming that the total Ca^2+^ remained unchanged during acidosis, the concentration of BAPTA that was required in the EGTA-BAPTA solution could be calculated as: 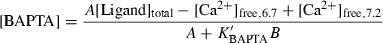

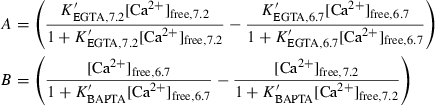


where 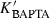 = 4.7 × 10^6^ m^−1^ is the apparent affinity constant of BAPTA at 36 °C and an ionic strength of 194 mm (Harrison & Bers 1987). 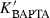 can be assumed to remain unchanged with acidification (Tsien 1980). 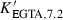
= 7.49 × 10^6^ m^−1^ is the apparent affinity constant of EGTA at pH 7.2, 37 °C and ionic strength of 150 mm (Harrison & Bers 1989). At pH 6.7 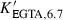 = 7.69 × 10^5^m^−1^. The EGTA concentration was subsequently calculated from [EGTA] = [Ligand]_total_ − [BAPTA] and total Ca^2+^ could then be determined. The total Mg^2+^ added to ensure a final concentration of 1 mm free Mg^2+^ in a setting of 5 mm ATP was calculated from (Ashley & Moisescu 1977): 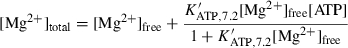


where 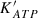 = 10^4^ m^−1^ is the apparent affinity constant of ATP for Mg^2+^ at pH 7.1, 37 °C (Stephenson & Williams 1981). Table 1 summarizes the pipette solutions.

**Table 1:** Composition of patch-clamp solutions used in the whole-cell patch-clamp experiments (mm)

	BAPTA	EGTA-BAPTA
K-aspartate	110	110
Na_2_-ATP	5	5
MgCl_2_	5.37	5.37
HEPES	10	10
EGTA		4.12
BAPTA	5	0.88
CaCl_2_	1	1.29
pH	7.2	7.2
Free Mg^2+^	1	1
Free Ca^2+^ (pH = 7.2)	5 × 10^−5^	5 × 10^−5^ (3 × 10^−4^ at pH 6.7)

### Imaging of cytosolic Ca^2+^

For confocal microscope imaging of intact myocytes, cells were pre-loaded with 10 μm Fluo-4-AM (20% Pluronic-DMSO) for 30 min followed by a 20 min wash. Cells were placed in a custom-designed experimental chamber placed on an inverted Leica confocal microscope (Model TCSSP5; Leica, Heidelberg, Germany). The chamber was perfused at 2–3 mL min^−1^ and the bathing solution could be completely exchanged within ∼2 min. Cells were stimulated by field stimulation at 0.5 Hz using constant voltage pulses (30 V cm^−1^, 2 ms duration). All recordings were done in frames (64 × 64) with a frame speed of ∼16 Hz. External solutions were similar to patch-clamp experiments. All experiments were performed at room temperature (∼24 °C). For quantification, the fluorescence in a cell-free region of the image (*F*_background,control_) was subtracted from the fluorescence of resting cells in control conditions (*F*_rest,control_). This gave the *F*_0_ value. Amplitudes of fluorescence signals during AP-evoked Ca^2+^ transients and during Ca^2+^ waves, *F*_peak,test_, were related to this *F*_0_ value after subtracting the resting fluorescence of the cell under the conditions when the transients or waves were recorded, *F*_rest,test_. 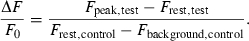


For imaging of patch-clamped cells, 100 μm cell-impermeant Fluo-4 was added to the pipette solution and the fluorescence was recorded using a Cairns photometric Optoscan system (Cairns Research, Faversham UK).

### Chemicals

All chemicals were of analytical grade. Fluo-4-AM, cell-impermeant Fluo-4 and pluronic DMSO were from Molecular Probes (Invitrogen, Carlsbad, CA, USA). Collagenase type II was from Worthington (Lakewood, NJ, USA). KN-93 was from Tocris (Bristol, Avon, UK); all other chemicals were from Sigma-Aldrich (Poole, Dorset, UK).

### Statistical analysis

Fisher’s exact test was used to assess whether association between two categorical data groups was non-random. Student’s *t*-test was used to test whether two data groups, which were assumed to be normally distributed, had identical means. All *t*-tests were preceded by *F*-tests to test for equal variance between two data groups concerned. Average data are presented as means ± SEM. *P* < 0.05 was considered the limit of significance.

## Results

### KN-93 suppresses spontaneous arrhythmogenesis during metabolic acidification

**Figure 1:**
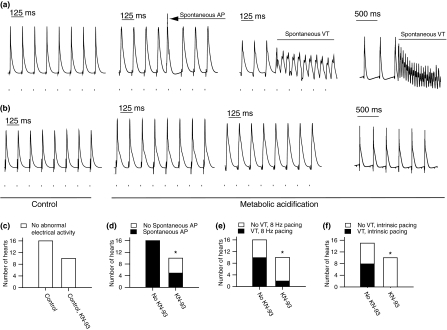
Effect of CaMKII inhibition on arrhythmogenicity of metabolic acidification. Representative recordings of epicardial MAPs from hearts perfused with control solution or solution causing metabolic acidification in the absence (a) or presence (b) of CaMKII inhibition (2 μm KN-93). Panels (a) and (b) show recordings during regular 8 Hz pacing and during intrinsic rhythm. Stimulations are indicated by squares under traces. No arrhythmic tendencies were observed during control conditions in the presence or absence of KN-93 (c). Panels (d)–(f) show the numbers of hearts that fired spontaneous APs (d), developed VT during regular pacing (e) and precipitated to VT during intrinsic rhythm (f) during metabolic acidification in the presence and absence of KN-93. *Significant difference between the absence and presence of KN-93 as assessed using Fisher’s exact test.

Arrhythmogenicity produced by metabolic acidification was initially assessed by recording epicardial MAPs from intact, Langendorff-perfused, hearts. All hearts were both initially examined under control conditions of normal extracellular pH and then during subsequent metabolic acidification. Figure 1a shows that during 8 Hz regular pacing metabolic acidification was associated with the appearance of spontaneously generated APs. In addition, ventricular tachycardia (VT) was observed both during 8 Hz regular pacing and during intrinsic rhythm. In contrast, inclusion of the CaMKII inhibitor KN-93 abolished VT during intrinsic rhythm and reduced the incidence of VT during regular pacing (Fig. 1b). Figure 1c–f quantifies these observations from 26 hearts exposed to metabolic acidification with the differing accompanying conditions. Of these, 16 hearts were studied in the absence and 10 in the presence of KN-93 using the protocols illustrated in Figure 1a,b. Abnormal electrical activity was not observed in any of these 26 hearts under the initial conditions of normal extracellular pH whether during regular pacing or intrinsic rhythm (Fig. 1c). However, acidified hearts subject to 8 Hz regular pacing showed evidence of spontaneous arrhythmogenic tendencies that could be classified into spontaneous firing of single APs or spontaneous VT (Fig. 1d,e respectively). Thus, in the absence of KN-93, acidification resulted in spontaneous APs in all hearts and spontaneous VT in 63% of the hearts. In the presence of KN-93, acidosis only produced spontaneous APs in 50% of the hearts and VT in 20% of the hearts (*P* < 0.01 and < 0.05, testing findings obtaining in the presence vs. the absence of KN-93 for spontaneous APs and spontaneous VT during metabolic acidosis respectively: Fisher’s exact test). During intrinsic rhythm, metabolic acidification was similarly associated with spontaneous VT in 53% of the hearts when KN-93 was absent (Fig. 1f). However, acidification did not induce spontaneous VT in intrinsically active hearts exposed to KN-93. During intrinsic rhythm CaMKII inhibition thus markedly reduced spontaneous arrhythmogenicity produced by acidosis (*P* < 0.01, presence vs. absence of KN-93 during acidosis) but CaMKII inhibition did not affect the frequency of the intrinsic rhythm under control or acidic conditions. Thus, under control conditions of normal extracellular pH, the intrinsic rhythm was 228 ± 64 beats min^−1^ and 197 ± 41 beats min^−1^ in the absence and presence of KN-93 respectively (*P* < 0.69). Under acidified conditions the intrinsic rhythm was 127 ± 35 and 197 ± 56 beats min^−1^ in the absence and presence of KN-93 respectively (*P* < 0.33). Taken together these findings suggest that CaMKII inhibition reduced spontaneous arrhythmogenicity caused by metabolic acidification whether during regular 8 Hz pacing or during intrinsic activity.

**Figure 2:**
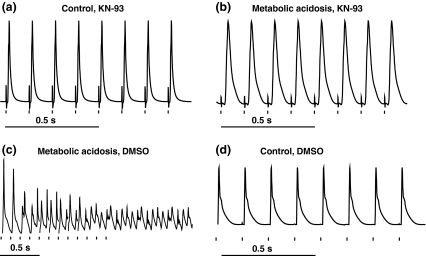
Reversibility of CaMKII inhibition on arrythmogenicity of metabolic acidification. Representative recordings of epicardial MAPs from a Langendorff-perfused murine heart paced regularly at 8 Hz during exposure to metabolic acidification in the absence and presence of KN-93. (a) Control conditions with KN-93, (b) metabolic acidification with KN-93, (c) metabolic acidification after removal of KN-93 and (d) control conditions without KN-93.

Complementary experiments on three hearts exposed to 8 Hz regular pacing showed that the anti-arrhythmic effects of KN-93 during metabolic acidosis were reversible. In these experiments KN-93 was initially introduced under conditions of normal pH (Fig. 2a) and during the following metabolic acidification that lasted 20 min (Fig. 2b). KN-93 was then removed, leaving the DMSO vehicle while metabolic acidification was maintained for another 20 min (Fig. 2c). After this 40 min of acidification, the control solution without KN-93 was re-introduced (Fig. 2d). In all hearts studied, VT during metabolic acidification only occurred after KN-93 was washed out.

### CaMKII-independent reductions in transmural repolarization gradients during metabolic acidification

Data of the kind illustrated in Figure 2csuggested an association between metabolic acidification and spontaneous and sustained arrhythmias under the conditions adopted in the present experiments. The occurrence of such arrhythmias during intrinsic rhythm or during regular pacing requires initiation by a triggering event. However, once triggered, continued arrhythmogenic activity requires maintenance by an arrhythmic substrate. Previous work in gain-of-function murine models for long QT syndrome 3 (LQT3) and hypokalaemia has attributed such arrhythmic substrate to reduced transmural repolarization gradients in the left ventricles resulting from preferential prolongations of epicardial over endocardial MAPs (Killeen *et al.* 2007, Thomas *et al.* 2008).

**Figure 3:**
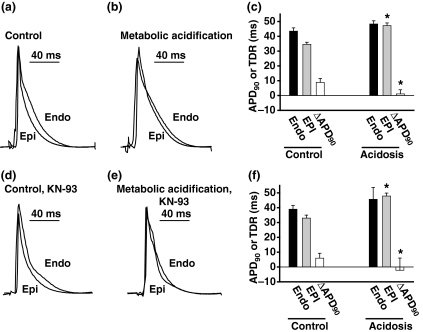
CaMKII inhibition reduces spontaneous arrhythmia during metabolic acidification but does not affect the reduction in transmural gradient of repolarization (ΔAPD_90_) during acidosis. Representative recordings of epicardial and endocardial MAPs during control conditions (a) and during metabolic acidification (b). Summarized data and calculated values for ΔAPD_90_ during control conditions and during metabolic acidification (c). Representative epicardial and endocardial MAPs in the presence of KN-93 during control conditions (d) and during metabolic acidification (e). Summarized data for experiments with KN-93 (f). *Significant difference between control conditions and metabolic acidification.

The experiments that followed tested for the possible relationships between such substrate, the arrhythmogenecity brought about by acidosis and possible contrasting effects of KN-93 upon these. They investigated the effects of metabolic acidification and KN-93 on left ventricular epicardial and endocardial MAPs in intact hearts during regular 8 Hz pacing. Figure 3a,b superimposes representative epicardial and endocardial MAP recordings obtained under conditions of normal extracellular pH (Fig. 3a) and during metabolic acidification (Fig. 3b). Figure 3d, e gives a similar display of recordings obtained in the presence of KN-93. MAP duration was assessed from the time to reach 90% repolarization (APD_90_). Such experiments demonstrated that metabolic acidification significantly prolonged epicardial APD_90_ regardless of whether KN-93 was absent (∼40%, *P* < 0.001, Fig. 3c) or present (∼44%, *P* < 0.001, Fig. 3f). In contrast, such acidification did not alter endocardial APD_90_ values whether KN-93 was absent (*P* = 0.14, Fig. 3c) or present (*P* = 0.47, Fig. 3f). In addition, KN-93 did not affect either epicardial or endocardial APD_90_ whether under conditions of normal extracellular pH (*P* = 0.55 and 0.27 respectively) or during metabolic acidification (*P* = 0.95 and 0.66 respectively).

These findings associate metabolic acidification with significantly reduced left ventricular transmural gradients of repolarization (ΔAPD_90_ = endocardial APD_90_ − epicardial APD_90_) but demonstrate that these were independent of CaMKII inhibition (*P* = 0.46, absence vs. presence of CaMKII).

### Metabolic acidification is associated with a CaMKII-dependent triggering of arrhythmia

**Figure 4:**
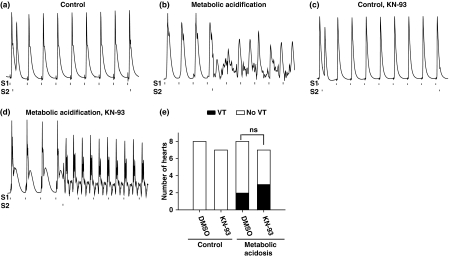
CaMKII inhibition does not affect the arrhythmogenic substrate arising during metabolic acidification. To assess for arrhythmic substrate the PES protocol was employed to stimulate the hearts with a regular 8 Hz pacing (S1) and with extrasystolic stimuli (S2), which are included to mimic the appearance of intrinsic arrhythmogenic triggers. (a) and (b) show epicardial MAP recordings during the PES stimulation in control conditions (a) and during metabolic acidification (b). During acidosis the S2 stimulus triggered VT. Similar representative traces from experiment performed in the presence of KN-93 are presented in (c) and (d). In panel (e) the summarized data are presented.

The above measurements of left ventricular ΔAPD_90_ appeared to exclude an involvement of arrhythmogenic substrate in effects of KN-93 under conditions of metabolic acidification. This notion was reinforced by the results of stimulating hearts using PES (Killeen *et al.* 2007, Thomas *et al.* 2008). PES involves a stimulation of hearts by external extrasystolic stimuli that mimic the appearance of intrinsic triggers for arrhythmia. PES is accordingly considered primarily to provide a means for assessing arrhythmic substrate that might *sustain*, rather than triggered activity that might *initiate*, arrhythmogenesis (Killeen *et al.* 2007, Thomas *et al.* 2008). The present experiments demonstrate that in contrast to findings obtained under conditions of normal extracellular pH (Fig. 4a), PES could induce VT under conditions of metabolic acidification (Fig. 4b) consistent with an appearance of the required arrhythmic substrate. In the presence of KN-93, PES similarly did not induce any abnormal electrical activity under conditions of normal extracellular pH (Fig. 4c). However, it did induce VT during metabolic acidification (Fig. 4d). Thus, during metabolic acidification PES caused VT in 25% of the hearts in the absence of KN-93 and in 43% of hearts when KN-93 was present (*P* = 0.61). The episodes of VT during metabolic acidification that were observed either using regular pacing or PES, generally had a monomorphic appearance with activation cycles of 76 ± 8 ms and 71 ± 7 ms in the absence and presence of KN-93 respectively. This indicates that KN-93 did not markedly affect the arrhythmic substrate during acidosis.

Taken together the whole heart experiments suggest that CaMKII inhibition acts to reduce AP *triggering* mechanisms without affecting the *arrhythmic substrate* that arises during metabolic acidification.

### Metabolic acidosis induces CaMKII-dependent Ca^2+^ waves

The cellular mechanisms behind the arrhythmogenic trigger during acidosis and the role of CaMKII in this triggering were next explored by confocal microscope imaging to compare SR Ca^2+^ handling both under conditions of normal extracellular pH and during metabolic acidification in the absence and presence of KN-93.

**Figure 5:**
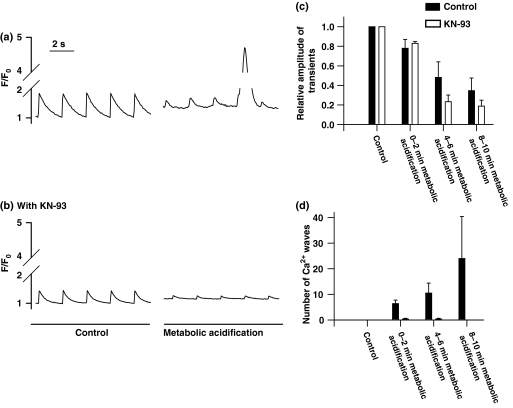
Effect of CaMKII inhibition on AP-evoked SR Ca^2+^ transients and SR Ca^2+^ waves during metabolic acidosis. (a, b) Representative 10 s recordings of AP-evoked SR Ca^2+^ transients (0.5 Hz) in control conditions (left panels) and after 5 min of metabolic acidification (right panels) in the absence (a) and presence of KN-93 (b). During acidification a Ca^2+^ wave was observed in the absence of KN-93. (c) Effect of KN-93 on amplitudes of AP-evoked SR Ca^2+^ transients during acidosis relative to those observed under control conditions and (d) the number of Ca^2+^ waves observed during 2 min sampling periods during control conditions and during acidosis. Average data are shown as mean ± SEM.

Figure 5a,b shows 10 s records of Ca^2+^ transients evoked by 0.5 Hz stimulation of typical ventricular myocytes in the absence (Fig. 5a) or presence (Fig. 5b) of KN-93 under conditions of normal extracellular pH (left panels) and during subsequent metabolic acidification (right panels). Under control conditions, KN-93 did not significantly affect the magnitudes of Ca^2+^ transients. First, metabolic acidification was associated with reduced amplitudes of the evoked Ca^2+^ transients in both the presence and absence of KN-93 (Fig. 5a,b). This decline in Ca^2+^ transients was most pronounced in the presence of KN-93 (Fig. 5c), which would be consistent with previous reports in which elevated CaMKII activity under acidotic conditions counteracted the inhibitory actions of acidosis on AP-evoked Ca^2+^ transients (Hulme *et al.* 1997, Komukai *et al.* 2001, DeSantiago *et al.* 2004). However, despite this decline, acidification also resulted in an appearance of large spontaneous Ca^2+^ waves (Fig. 5a, right panel; see also Fig. 5d). Remarkably, these waves were abolished by KN-93: Less than 1 wave was seen on average during 4–6 min of acidification in the presence of KN-93 whereas more than 10 waves were seen in its absence. Such Ca^2+^ waves are likely to play a substantial role for triggering spontaneous APs during acidosis by potentially generating delayed after depolarizations (DADs).

Secondly, as reported in other studies, acidification was associated with a CaMKII-independent increase in the free cytosolic Ca^2+^ (Orchard *et al.* 1987, Hulme *et al.* 1997). Thus, after 5 min of acidification this baseline Ca^2+^ had increased by 38 ± 18% in the absence of KN-93 and by 22 ± 11% in the presence of KN-93 (*P* = 0.62).

### Metabolic acidification causes CaMKII-dependent oscillations in the resting membrane potential and spontaneous APs

**Figure 6:**
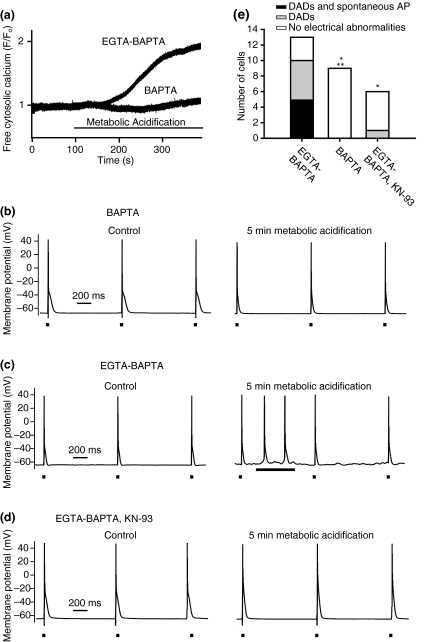
Effect of cytosolic free Ca^2+^ and KN-93 on single cell APs in ventricular myocytes. Recordings of single cell APs (1 Hz) were performed using the whole-cell patch-clamp configuration. APs were initially recorded under control conditions and, subsequently, after 5 min of metabolic acidification. Squares indicate times of stimulation. (a) Representative traces of baseline free Ca^2+^ during control conditions and during the first 5 min of acidosis using EGTA-BAPTA solution or BAPTA solution. (b–d) Representative AP recordings using BAPTA solution (b), EGTA-BAPTA solution (c) and EGTA-BAPTA solution with KN-93 (d). Records have not been corrected for junction potentials of 17.3 and 13.6 mV for control and acid solution respectively. (e) The proportions of acidified cells that developed (1) DADs and spontaneous APs, (2) only DADs or (3) no abnormal electrical activity under the three conditions shown in (b)–(d). *Significantly smaller proportion of acidified cells developed DADs than the acidified cells patched-clamped using EGTA-BAPTA solution and in the absence of KN-93; **Fisher’s exact test.

Finally, to test whether such DADs and spontaneous APs during metabolic acidification could indeed be observed at the cellular level, single cell APs were recorded using whole-cell patch-clamp recordings from ventricular myocytes. The confocal microscopy recordings showed that in accordance with previous studies, acidification was associated with a CaMKII-independent elevation in free cytosolic Ca^2+^ (Fig. 5a,b) and it has been suggested that increased CaMKII activity during acidosis arises secondarily to this increased free cytosolic Ca^2+^ (Hulme *et al.* 1997). In our experiments, this increase in Ca^2+^ was mimicked by using patch-clamp electrodes filled with the EGTA-BAPTA solution designed to allow free Ca^2+^ to rise from ∼50 to ∼300 nm for a drop of pH from 7.2 to 6.7. Results obtained using this pipette solution were compared with those obtained using electrodes containing a BAPTA solution that should prevent major alterations in free Ca^2+^ with imposed acidification. To test whether free Ca^2+^ was indeed differently affected by imposed acidification with the use of the two pipette solutions, the baseline free Ca^2+^ was initially measured in patch-clamped cells during the first 5 min of acidification. Figure 6a shows representative traces of the free Ca^2+^ from such experiments. In all six cells that were patch-clamped with the EGTA-BAPTA solution, three of which were treated with KN-93, acidification caused a marked change in the free Ca^2+^. In contrast, with the BAPTA solution the acidic condition did not markedly affect free Ca^2+^. In addition to confirming that only cells that were patch-clamped using EGTA-BAPTA solution showed significant changes in free Ca^2+^ during acidosis, these experiments also confirmed that a net *intracellular* acidification was achieved following imposition of *extracellular* acidification.

Figure 6b–d shows records of APs obtained at a stimulation frequency of 1 Hz under conditions of normal extracellular pH (left panels) and after 5 min of subsequent metabolic acidification (right panels) of cells patch-clamped using either the BAPTA (Fig. 6b,e) or the EGTA-BAPTA solution (Fig. 6c,d). In cells investigated using BAPTA solution, metabolic acidification did not cause electrophysiological abnormalities in the nine cells investigated (Fig. 6b). In contrast, in cells patch-clamped using the EGTA-BAPTA solution, acidification caused oscillations in the resting membrane potential (10 of 13 cells) that occasionally led to episodes of spontaneous APs (5 of 13 cells) (Fig. 6c,e). However, when experiments were performed using EGTA-BAPTA solution and in the presence of KN-93, resting membrane potential oscillations during acidosis were only observed in one of six cells and spontaneous APs were not observed in any of the cells examined (Fig. 6d,e).

In cells investigated with the BAPTA solution the maximal rate of rise in the APs (d*v*/d*t*) dropped from 530 ± 43 V s^−1^ before acidification to 426 ± 36 V s^−1^ after 5 min acidification (*P* < 0.01, *n*=7). However, the APD_90_ remained unchanged (35 ± 6 and 29 ± 7 ms; *P* = 0.42, *n*=7). In the experiments with EGTA-BAPTA solution the presence of KN-93 did not affect either d*v*/d*t* or APD_90_ under control conditions or during acidosis. Thus, d*v*/d*t* dropped from 503 ± 48 to 476 ± 24 V s^−1^ after 5 min acidification in the absence of KN-93 (*P* = 0.43, *n*=8) and from 492 ± 71 to 440 ± 85 V s^−1^ in the presence of KN-93 (*P* = 0.65, *n*=5), also suggesting an absence of significant effects of KN-93 by itself on d*v*/d*t* either before or after acidification. With EGTA-BAPTA solution APD_90_ was 51 ± 6 ms under control conditions and 40 ± 7 ms after 5 min of metabolic acidification in the absence of KN-93 (*P* = 0.23, *n*=8) and in the presence of KN-93 APD_90_ was 65 ± 12 ms under control conditions and 43 ± 9 ms after 5 min of metabolic acidification (*P* = 0.16, *n*=5).

## Discussion

Metabolic acidification is a key cellular alteration in a number of clinically important situations including the cardiac ischaemia that occurs with acute coronary artery occlusion. Such situations are associated with increased arrhythmogenicity (Senges *et al.* 1979, Elliott *et al.* 1992, Vandenberg *et al.* 1993, Luqman *et al.* 2007). This study demonstrates for the first time that in murine hearts the arrhythmia occurring during such metabolic acidification can be triggered via CaMKII-dependent mechanisms in turn involving alterations in cellular Ca^2+^ homeostasis. It reports experiments that assessed acute arrhythmogenicity in intact Langendorff-perfused hearts and correlated these findings with corresponding changes in electrical activity and Ca^2+^ homeostasis at the single cell level. Both types of experiments involved a measurement of physiological properties in the absence and presence of metabolic acidification achieved by lactic acid administration. This is known to produce a rapid intracellular acidification via its transmembrane transport by monocarboxylate transporters (Poole *et al.* 1989). The experiments at the *whole heart level* were performed to characterize the arrhythmia arising during metabolic acidification while experiments at the single cell level were employed to relate these findings to earlier cellular studies focusing on the effect of metabolic acidification on Ca^2+^ homeostasis. As such, three aspects of the arrhythmia arising during metabolic acidification were explored in this study; (1) the enhanced arrhythmic triggering, (2) the re-entrant substrate and finally (3) the role of CaMKII in the arrhythmogenecity associated with metabolic acidification.

First, the experiments at the *whole heart level* separated arrhythmic tendency during metabolic acidification that could be attributed to *triggered activity* from arrhythmic tendency arising from *re-entrant substrate* as detected using PES stimulation. During intrinsic rhythm a significant proportion of acidified hearts developed spontaneous VT. With regular 8 Hz pacing, all acidified hearts fired spontaneous APs and a significant proportion showed VT. These findings associated metabolic acidification with the appearance of spontaneous APs that could act as *triggers* for VT. To study the role of SR Ca^2+^ handling in this arrhythmic triggering during metabolic acidification confocal imaging of intact myocytes was performed during initial conditions of normal extracellular pH and subsequently during metabolic acidification. These experiments directly demonstrated that acidosis led to the appearance of large Ca^2+^ waves, known to cause DADs via an increased Na^+^-Ca^2+^ exchange current (Coetzee & Opie 1987, Orchard & Cingolani 1994, Dibb *et al.* 2007). The large amplitudes of the Ca^2+^ waves were compatible with a markedly increased SR Ca^2+^ content during acidification. The latter would agree with other studies focusing on the effect of acidification on SR Ca^2+^ loading (Orchard *et al.* 1987, Orchard & Cingolani 1994). It is therefore likely that the spontaneous arrhythmia in the whole heart during metabolic acidification resulted from SR Ca^2+^ waves and DADs. Such a notion is further supported by the patch-clamp experiments, which could study spontaneous electrical activity in single isolated ventricular myocytes in isolation from re-entrant substrate. Results were compared between cells patch-clamped using pipettes that contained either BAPTA solution or EGTA-BAPTA solution. In the first case, metabolic acidification altered neither cytosolic free [Ca^2+^], reflected in the fluorescence of a loaded Fluo-4, nor the normal pattern of AP firing, in so doing additionally excluding significant arrhythmogenic actions of the lactate ion itself in the present experiments. In the second case using the EGTA-BAPTA solution, metabolic acidification increased intracellular Ca^2+^ similar to the rise in baseline Ca^2+^ in the confocal imaging during acidosis, in so doing confirming successful cell acidification, and produced DADs and spontaneous APs. The above findings together suggest *in vivo* situations in which acidosis elevates free cytosolic Ca^2+^ that in turn produces altered SR Ca^2+^ handling and triggered arrhythmic phenomena.

Secondly, in addition, metabolic acidification also resulted in re-entrant substrate that might result in persistent arrhythmogenesis following such spontaneous APs. Thus, epicardial and endocardial MAP recordings suggested an association between metabolic acidification and reduced ΔAPD_90_ arising from preferential prolongation of epicardial MAPs. This is in common with the re-entrant arrhythmic substrate reported in murine models of LQT3 and hypokalaemia (Killeen *et al.* 2007, Thomas *et al.* 2008). It has previously been shown in Langendorff-perfused whole hearts that arrhythmic tendencies arising during acidification can be reduced by a gap junction opener (Eloff *et al.* 2003), indicating that during acidosis reduced conduction velocity may generate an arrhythmic substrate separate from the reduced ΔAPD_90_. This is further supported by the reduced d*v*/d*t* of the single cell APs during acidosis, which indicates that acidification caused a reduction in Na^+^ current during the AP that may add to closure of gap junctions in generating a reduced AP conduction velocity.

Thirdly, acidification has also been associated with elevated CaMKII activity (Hulme *et al.* 1997, Komukai *et al.* 2001, DeSantiago *et al.* 2004). Chronic CaMKII overexpression is known to cause cardiac hypertrophy and increased arrhythmogenicity in murine hearts (Wu *et al.* 2002). Also, a recent study demonstrated that CaMKII is involved in arrhythmogenecity after a period of respiratory acidosis (Said *et al.* 2008). However, the role of CaMKII in the generation of acute arrhythmogenecity under acidotic conditions has not been investigated. In the present experiments, in acidified Langendorff-perfused hearts, KN-93 abolished all evidence of VT during intrinsic rhythm and sharply reduced the incidence of spontaneous APs and VT during regular pacing. Previous work has indicated that KN-93 elicits potent and specific inhibitory effects on CaMKII phosphorylating activity similar to those of the very specific peptide inhibitor AIP (Sumi *et al.* 1991, Kohlhaas *et al.* 2006, Vila-Petroff *et al.* 2007). Furthermore, in patch-clamped single cells, CaMKII inhibition produced either by KN-93 or indirectly by avoidance of a rise in cytosolic Ca^2+^ during acidosis greatly reduced DADs and completely abolished spontaneous APs during acidosis. In confocal imaging experiments, KN-93 caused a larger decline in the electrically evoked Ca^2+^ transients and abolished the spontaneous SR Ca^2+^ waves during the metabolic acidification despite acidosis still causing a marked rise in baseline Ca^2+^ in the presence of KN-93. However, it did not prevent the arrhythmias and abnormal electrical activity during PES. Nor did it affect epicardial or endocardial APD_90_, or ΔAPD_90_ whether during control conditions or during metabolic acidification. Together these findings indicate that whilst CaMKII did not affect the substrate for arrhythmia that arose with metabolic acidification, it enhanced AP triggering during metabolic acidosis by the generation of spontaneous SR Ca^2+^ waves.

Spontaneous SR Ca^2+^ waves are generally believed to occur when the free luminal SR Ca^2+^ reaches a threshold concentration for RyR2A opening (Dibb *et al.* 2007), following enhanced SR Ca^2+^ uptake or reduced thresholds for RyR2A opening. The increased phosphorylation of the CaMKII-specific site on phospholamban during acidosis (Hulme *et al.* 1997, DeSantiago *et al.* 2004) is believed to relieve the inhibitory effect of phospholamban on SERCA. Indeed, it is well established that KN-93 can suppress the enhanced SR Ca^2+^ content usually observed during acidosis (Komukai *et al.* 2001, DeSantiago *et al.* 2004). CaMKII-mediated RYR2a phosphorylation may also increase RyR2a opening probabilities (Guo *et al.* 2006), effectively lowering the threshold concentration of free SR Ca^2+^ that leads to RyR2A opening. However, a recent study suggested that CaMKII phosphorylation of RyR2A actually reduces its opening probability (Yang *et al.* 2007). Thus, we speculate that the Ca^2+^ waves observed in this study in metabolically acidified myocytes could arise from SR Ca^2+^ overloading due to CaMKII-mediated phospholamban phosphorylation and possibly from CaMKII-mediated RYR2a phosphorylation causing increased opening probability of RyR2a.

It is well established that the activity of CaMKII becomes elevated during structural heart disease (Yang *et al.* 2005) and during ischaemia/reperfusion (Vila-Petroff *et al.* 2007). Experiments with animal models of cardiac hypertrophy have implicated CaMKII in the structural remodelling and arrhythmogenesis associated with cardiac hypertrophy (Wu *et al.* 2002). Also, in mice where hypertrophy was induced by chronic adrenergic stimulation, treatment with KN-93 or expression of a CaMKII inhibitory peptide, AC3-I, successfully reduced structural remodelling and arrhythmogenecity (Yang *et al.* 2005). These studies implicate chronically elevated CaMKII activity in heart disease. In the present study, acute metabolic acidification caused CaMKII-dependent increases in ventricular automaticity. As metabolic acidification is a hallmark of acute cardiac ischaemia, we suggest that CaMKII may be involved in the triggering of cardiac arrhythmia during acute myocardial ischaemia.

## Conflict of interest

We report no conflict of interest.
